# Decoding Glioblastoma Complexity Through Extracellular Vesicles, Organ-on-Chip Models, and Deep Learning

**DOI:** 10.3390/cells15121080

**Published:** 2026-06-14

**Authors:** Domenico Amato, Giuseppa D’Amico, Salvatore Calderaro, Alessandra Maria Vitale, Pierlorenzo Veiceschi, Francesco Cappello, Celeste Caruso Bavisotto, Giosuè Lo Bosco

**Affiliations:** 1 Department of Mathematics and Computer Science, University of Palermo, Via Archirafi 34, 90123 Palermo, Italy; domenico.amato01@unipa.it (D.A.); salvatore.calderaro01@unipa.it (S.C.); 2 Section of Human Anatomy and Histology, Department of Biomedicine, Neuroscience and Advanced Diagnostics (BIND), University of Palermo, Via del Vespro 129, 90127 Palermo, Italy; giuseppa.damico01@unipa.it (G.D.); alessandramaria.vitale@unipa.it (A.M.V.); pierlorenzomaria.veiceschi@unipa.it (P.V.); francesco.cappello@unipa.it (F.C.); 3 Department of Physics and Chemistry, University of Palermo, Via Archirafi 36, 90123 Palermo, Italy; 4 Euro-Mediterranean Institute of Science and Technology (IEMEST), Via Michele Miraglia, 90139 Palermo, Italy; 5 Department of Neurosurgery, National Relevance and High Specialization Hospital Trust ARNAS Civico, 90127 Palermo, Italy

**Keywords:** glioblastoma, extracellular vesicles, exosomes, organ-on-chip, tumor microenvironment, blood–brain barrier, deep learning, microfluidics, 3D in vitro models, precision oncology

## Abstract

Glioblastoma (GBM) is one of the most aggressive human cancers, with therapeutic failure driven by pronounced intratumoral heterogeneity, microenvironmental plasticity, immune suppression, blood–brain barrier (BBB)-related pharmacological constraints, and adaptive resistance mechanisms. A major limitation in GBM research is the lack of a human-relevant experimental system able to reproduce these dynamic features while generating interpretable, multimodal datasets. In this context, we propose a testable organ-on-chip (OoC)-extracellular vesicle (EV)-deep learning (DL) framework in which patient-derived GBM cells, endothelial cells, astrocytes, pericytes, stromal cells, and immune components are organized within perfused microphysiological systems. EVs are selectively and temporally harvested from defined compartments, and imaging, barrier-function, sensor, and EV-cargo data are integrated through modality-specific and multimodal DL architectures. This framework is intended not as an immediately validated clinical tool but as an experimental roadmap for linking EV-mediated communication to measurable phenotypes such as BBB disruption, invasion, immune reprogramming, and drug response. We critically discuss the technical requirements of BBB-on-chip systems, EV source attribution, immune-component integration, DL model selection, data scarcity, overfitting, batch effects, domain shift, regulatory barriers, cost, throughput, and reproducibility. By repositioning OoC-EV-DL integration as a staged translational strategy rather than a clinically established solution, this work aims to define a realistic and biologically grounded route for advancing precision oncology in GBM.

## 1. Introduction: The Need for Innovation in Glioblastoma (GBM) Research

GBM is the most aggressive and malignant primary brain tumor in adults. It is characterized by rapid growth, brain infiltration, high cellular heterogeneity, and a median overall survival that remains approximately 12–18 months despite standard-of-care therapy [1,2,3,4]. The current standard treatment includes maximal safe surgical resection followed by radiotherapy and temozolomide (TMZ)-based chemotherapy, which improves progression-free and overall survival but is rarely curative [3]. These unfavorable outcomes are partly attributable to GBM stem cells (GSCs), which contribute to tumor initiation, progression, therapy resistance, and recurrence [5]. Another key contributor to therapy resistance is the highly immunosuppressive tumor microenvironment (TME), which represents a major obstacle to effective immunotherapy [6]. The main components of the TME include myeloid-derived suppressor cells (MDSCs), glioma-associated macrophage/microglia (GAM), and regulatory T cells (Tregs), which together create a complex, self-reinforcing immunosuppressive network [7,8,9]. These biological features contribute to the highly malignant and treatment-resistant nature of GBM, highlighting the need for experimental systems able to connect tumor-intrinsic molecular states, microenvironmental interactions, and therapy-induced adaptation within a unified and testable framework. In this context, the development of robust preclinical models that faithfully recapitulate the biological complexity and heterogeneity of GBM and its dynamic TME is critical for the design of more effective, targeted, and personalized therapeutic strategies [10,11].

Conventional two-dimensional (2D) cell cultures have been used to investigate GBM biology and underlying mechanisms [12,13]. However, they do not fully replicate the complex and dynamic TME, particularly concerning cell-to-cell and cell-to-extracellular matrix (ECM) interactions.

Recently, three-dimensional (3D) cell culture models, including organoids and spheroids, have emerged as valuable tools in cancer research, offering numerous advantages compared to traditional 2D culture systems. In fact, these models better mimic the spatial organization and functional characteristics of in vivo tumors, allowing for more accurate investigation of tumor behavior and drug response [14,15,16]. However, they still have limitations and do not encompass the full complexity of TME, as they lack key components such as immune cells and vasculature [17].

To overcome these drawbacks, innovative bioengineering methods have been explored by combining tumor organoids, patient-derived cultures, and microfluidic devices, namely organ-on-chip (OoC) platforms [18]. In this review, the term preclinical model is used primarily to refer to in vitro and in silico new approach methodologies, rather than in vivo animal models. OoC platforms more closely mimic physiological environments, including vascularization, controlled oxygen and nutrient gradients, perfusion, and mechanical cues characteristic of the in vivo TME [19]. These features enable the investigation of tumor behavior under dynamic and variable conditions, including responses to therapeutic interventions and mechanisms underlying immunosuppressive states [19,20,21,22]. In addition, they allow the study of cell-to-cell communication mediated by extracellular vesicles (EVs) [23,24], which play a central role in shaping the GBM-surrounding microenvironment to promote tumor progression, immune evasion, and survival [25,26,27,28].

A complementary approach increasingly used in oncology involves deep learning (DL) and machine learning (ML) [29]. These technologies can analyze complex, high-dimensional biomedical data that are difficult to interpret with classical methods, including MRI, histopathology, live-cell imaging, genomics, transcriptomics, proteomics, and EV-derived multi-omics datasets [30,31,32]. However, their application to GBM precision oncology remains constrained by limited annotated datasets, inter-institutional domain shifts, batch effects, computational bias, and the need for transparent validation before clinical implementation.

The integration of these technologies with microfluidic cell culture systems and OoC devices holds significant potential for the rapid analysis of complex, high-dimensional datasets generated by these platforms [33]. Nevertheless, OoC systems should not be considered static models. Their value lies precisely in their ability to reproduce dynamic processes such as flow, perfusion, migration, barrier modulation, and temporal drug responses, although these dynamics are generally captured within defined experimental windows. Similarly, EV-derived data provide molecular snapshots at selected time points rather than continuous dynamic readouts. The conceptual advantage of the proposed framework is therefore not that OoCs are static and EVs are dynamic but that DL can integrate spatially resolved OoC-derived phenotypes with longitudinal EV-derived molecular information to identify relationships that are not accessible when either modality is analyzed independently.

Therefore, this work aims to move beyond a parallel description of EVs, OoC systems, and DL by proposing a logical pipeline:

- Reconstruct patient-relevant GBM niches on chip, including BBB and immune components;
- Perturb these systems with defined microenvironmental or therapeutic stimuli;
- Collect compartment-specific EVs and functional OoC readouts over time;
- Profile EV cargo and chip-derived imaging/barrier/sensor data;
- Use DL models to link EV-mediated communication with phenotypic outcomes such as invasion, immune suppression, BBB disruption, and treatment response.

The translational goal is not to claim immediate clinical readiness but to define a stepwise roadmap for experimental validation, biomarker prioritization, and eventual patient-stratification studies.

## 2. Brief Overview of Extracellular Vesicles (EVs)

EVs are released by virtually all cell types and represent a fundamental mechanism of intercellular communication in both physiological and pathological contexts [34,35,36,37]. They are characterized by a phospholipid bilayer that encapsulates a diverse cargo of bioactive molecules, including proteins, lipids, and nucleic acids, which reflect the molecular state of the originating cell [38,39,40,41]. Importantly, this lipid bilayer protects its content from enzymatic degradation, enabling stable transport through biological fluids such as blood, cerebrospinal fluid, and saliva [42,43,44,45,46]. Based on their biogenesis, size, density, and physicochemical properties, EVs have traditionally been classified into distinct subtypes, including exosomes, microvesicles, and apoptotic bodies [47,48]. However, due to the significant overlap in size ranges and molecular composition, as well as the absence of specific and universally accepted markers to unambiguously distinguish these subpopulations, the International Society for Extracellular Vesicles (ISEV) recommends the use of the generic term “extracellular vesicles (EVs)” and encourages their characterization based on experimentally measurable parameters, including size (e.g., small EVs < 100–200 nm and medium/large EVs > 200 nm), density and biochemical composition [49,50].

EVs are now recognized as key mediators of cell–cell communication across short and long distances, contributing to the regulation of tissue homeostasis as well as to disease progression [51,52,53]. In cancer, and particularly in GBM, EVs play a crucial role in decoding tumor complexity by dynamically transferring oncogenic signals between tumor cells and the surrounding microenvironment [54,55]. Through this continuous molecular exchange, GBM-derived EVs actively modulate multiple hallmarks of malignancy, including immune evasion, angiogenesis, invasion, and therapeutic resistance [56,57,58,59]. Moreover, EVs mirror the heterogeneity of their cells of origin, providing a real-time snapshot of tumor evolution and microenvironmental adaptation. This property makes them especially valuable not only as functional effectors of tumor progression but also as minimally invasive biomarkers accessible through liquid biopsy [60].

Within integrative experimental frameworks, such as OoC systems, EVs offer a unique opportunity to capture the dynamic dimension of GBM biology, complementing spatial and structural information with temporally resolved molecular communication.

In GBM, several EV-associated cargos have direct biological relevance. EGFRvIII-containing EVs can transfer oncogenic signaling to recipient cells; miR-21 and miR-10b are repeatedly implicated in proliferation, invasion, immune modulation, and therapy resistance; mutant IDH1-related molecular signatures may support glioma classification and monitoring; and EV-associated PD-L1 contributes to immune evasion through the suppression of antitumor immune responses [21,54,55,56,57,58,59,60,61,62]. These examples better illustrate why EVs should be treated not only as biomarkers but also as functional mediators of GBM-TME crosstalk.

A major technical limitation is source attribution. EVs recovered from plasma, cerebrospinal fluid, or complex culture systems represent heterogeneous vesicle populations released by tumor cells, endothelial cells, astrocytes, microglia, infiltrating immune cells, and stromal components. Distinguishing tumor-derived EVs from stromal or immune-derived vesicles therefore requires enrichment strategies, orthogonal characterization, and careful interpretation. In OoC platforms, compartmentalization, lineage labeling, selective outlet collection, and time-resolved sampling may reduce this ambiguity. When coupled with high-throughput molecular profiling and DL approaches, EV-derived data can be used to extract multidimensional signatures associated with tumor states and transitions, but these models require rigorous validation to avoid overinterpreting signals that may reflect culture conditions, batch effects, or non-tumor vesicle contamination [44,63,64,65].

## 3. The Emergence of Organ-on-Chip Models in Cancer Research

Organs-on-chips (OoCs) are an innovative technology designed to replicate selected structural and functional features of human organs on a miniaturized scale. These microengineered devices integrate living cells into precisely controlled microenvironments by means of microfluidic systems and bioengineering techniques [66,67]. This allows researchers to mimic key physiological and pathological processes more accurately than traditional in vitro models, such as 2D cell cultures, while reducing some limitations of animal models, which often fail to fully predict human responses because of interspecies differences [68,69]. As a result, this new technology is rapidly gaining attention in biomedical research, offering valuable insights into disease mechanisms and progression. Moreover, its importance also lies in the fact that it provides a powerful platform for testing new drugs and developing personalized therapeutic strategies, potentially reducing dependence on animal models and accelerating the time-course from discovery to clinical application [70,71].

A primary field of application for OoCs is cancer research, since one of the major challenges in oncology is the accurate modeling of the TME, which plays a crucial role in tumor development and progression, as well as in the formation of metastasis and response to therapies [72]. Traditional models often fail to reproduce the complex interactions between tumor cells and the surrounding environment, such as immune cells, extracellular matrix, and vascular components. In contrast, OoCs offer a more faithful and dynamic reproduction of the TME due to integrated microfluidic systems and incorporated sensors that enable precise control and real-time monitoring of key parameters (such as oxygen levels, pH, or the presence of metabolites), which significantly improve the study of tumor biology and drug response [73,74]. In the context of GBM, these systems can incorporate tumor cells (including patient-derived glioma stem cells), endothelial cells to model the vasculature, and, when required, stromal and immune components such as astrocytes, microglia, or peripheral immune cells. Compared to conventional 2D cultures, which lack three-dimensional organization and dynamic signaling, and to static 3D models that do not fully recapitulate perfusion or vascular interfaces, OoC systems enable the reconstruction of critical GBM features such as the perivascular niche, BBB interactions, and diffusion-limited gradients. This allows not only the study of tumor cell behavior under physiologically relevant conditions but also the investigation of processes that are difficult to capture in traditional models, including real-time cell migration along vascular structures, modulation of endothelial integrity, immune–tumor interactions, and the spatiotemporal dynamics of EV-mediated communication. Beyond their structural and cellular fidelity, OoC platforms provide a unique opportunity to investigate intercellular communication mechanisms that critically regulate tumor progression and therapy response. Among these mechanisms are EVs, which are increasingly emerging as key mediators of cell–cell and cell-microenvironment interactions in cancer, as they transfer bioactive cargo influencing angiogenesis, immune evasion, invasion, and therapeutic resistance [75]. Microfluidic architectures enable precise control of fluid flow, shear stress, and spatial compartmentalization, closely mimicking in vivo conditions that regulate EV release, transport, and uptake. Unlike static culture systems, OoCs allow real-time monitoring of EV-mediated signaling under dynamic perfusion, facilitating the study of EV trafficking across biological barriers such as the BBB [76,77]. This feature is especially relevant for GBM, where tumor-derived EVs may modulate vascular integrity, immune-cell polarization, and perivascular invasion [21,25,26,27,28,78,79,80]. In particular, BBB-on-chip models are highly relevant for GBM due to the role of the BBB and the blood–tumor barrier in drug delivery and treatment failure. A functional BBB-on-chip model typically describes the cellular components of the vascular side, such as brain microvascular endothelial cells, astrocytes, and pericytes, and can also include tumor cells or GSCs in a separate compartment for barrier disruption by tumors. The integrity of the barrier can be measured by transepithelial electrical resistance (TEER), permeability assays for fluorescent tracers or drugs, and tight-junction markers, as well as endothelial remodeling in a microfluidic device. Compared with conventional Transwell or static BBB models, microfluidic BBB-on-chip systems better reproduce shear stress, perfusion, and spatial organization, but they remain limited by differences in cell source, matrix composition, flow regime, and endpoint standardization [64,81,82].

A PubMed query performed on 25 May 2026 using the term “(organ-on-chip) AND (glioblastoma)” retrieved only 37 publications, underscoring the limited exploration of this topic to date. Although the 2017 report by Phan et al. remains relevant for BBB-on-chip modeling [82], an earlier BBB-on-chip study by Griep et al. demonstrated a microfluidic platform capable of mechanically and biochemically modulating BBB function [64]. Subsequent GBM-on-chip studies have recreated the perivascular niche, modeled migration toward chemoattractants, incorporated patient-derived GSCs, and enabled nanomedicine testing. For example, cubosomes—which are lipid-based, bicontinuous cubic-phase nanoparticles—have been functionalized to enhance BBB penetration and GBM targeting [83,84,85,86,87,88,89].

In conclusion, OoCs are powerful and rapidly evolving tools that have the potential to bridge the gap between traditional preclinical models and human pathophysiology, as they enable faithful reproduction of key features of GBM or its microenvironment, such as the PVN, the BBB, hypoxia, and cell–cell and cell-ECM interactions. Their application offers significant advances not only in understanding the complex biology of GBM (such as invasion mechanisms, stemness, and resistance to therapy) but also in the development and preclinical validation of targeted therapeutic strategies, ranging from chemotherapies and immunotherapies to nanomedicine-based approaches. Overall, OoC models can reproduce selected aspects of the GBM niche, but their translational value depends on standardized reporting of cellular components, flow conditions, matrix properties, barrier integrity metrics, and drug-permeability endpoints.

## 4. Integrating Deep Learning into Organ-on-Chip Systems for GBM

Despite significant progress in GBM research, traditional preclinical models, such as 2D cell cultures or animal models, often fail to reproduce the structural, cellular, and molecular complexity of the human brain microenvironment, which plays a key role in GBM pathogenesis and treatment resistance [90,91].

Conversely, OoC systems are capable of generating large volumes of multimodal data through the use of time-lapse microscopy, high-content imaging, embedded sensors, barrier-permeability measurements, and molecular profiling. For this reason, different DL architectures can be leveraged to analyze different data types [92]. For instance, convolutional neural networks, including U-Net-like segmentation, are well-suited for microscopy histopathology, MRI, spheroid segmentation, cell tracking, and morphological quantification [93]. Graph neural networks (GNNs) can be used to represent and model cell–cell interaction networks, spatial neighborhoods, molecular networks, and EV-mediated signaling relationships. Recurrent neural networks (RNNs) and transformers can be helpful for modeling longitudinal images, sensor streams, and multi-omics time series. In particular, multimodal transformers and cross-attention models are particularly suitable when imaging, clinical, molecular, and EV-derived data are used [94,95,96,97,98,99,100]. In a unified end-to-end OoC-EV-DL framework, imaging data from GBM-on-chip experiments could first be processed by a CNN to detect and quantify invasion morphology, cell death, vascular remodeling, and immune-cell migration. EV-derived miRNA, protein, DNA, methylation, or surface-marker profiles could instead be processed using classical dense neural networks or more complex networks such as variational autoencoders or sequence-based models. Given their ability to model relationships, GNN models could be useful to capture and encode relationships between tumor cells, endothelial cells, astrocytes, pericytes, GAMs, MDSCs, Tregs, and EV-cargos, while an attention-based fusion model could identify which modality most strongly contributes to specific outputs, such as barrier breakdown, immune suppression, or drug resistance. This structure makes the framework testable, meaning that a model prediction should be evaluated against measurable chip outputs and independently replicated datasets, as well as mechanistic perturbations such as EV depletion, pathway inhibition, or immune-cell exclusion.

Despite these advances, several computational challenges must be addressed to fully integrate DL into GBM-on-chip platforms. A primary limitation is the limited availability of data, together with its intrinsic heterogeneity and the difficulties associated with obtaining reliable annotations. Indeed, image acquisition protocols and cell sources may vary substantially across laboratories, while omics datasets are often affected by batch effects. Another major challenge concerns model generalization: models trained in a specific institutional setting frequently exhibit reduced performance when deployed in different environments due to domain shift and out-of-distribution (OOD) data.

Another risk is overfitting when high-dimensional molecular data are combined with data from limited patient-derived samples. To overcome these limitations, potential strategies may include predefined endpoints, external validation, transfer learning, few-shot learning, self-supervised pretraining, batch-effect correction, uncertainty quantification, ablation testing, and eXplainable Artificial Intelligence (XAI) methods such as saliency maps, SHAP values, attention visualization, and graph-based interpretability [97,98,99,100,101,102,103,104,105,106,107,108,109]. In this context, metric learning and graph-based approaches may improve interpretability in biomedical image classification. Metric learning can structure the embedding space according to semantic relationships between image features, enabling more compact and interpretable representations of medical images. When combined with baseline classifiers such as k-nearest neighbors or support vector machines, or with clustering algorithms such as fuzzy c-means and self-organizing maps, these approaches may support visual analytics, semantic similarity assessment, confidence scoring, and information-granule-based interpretation [103,104,105,106,107,108]. Similarly, graph neural networks can capture spatial and relational dependencies among image regions, potentially improving model transparency and decision traceability in complex GBM-on-chip imaging pipelines [108,109]. These methods are particularly relevant for patient-specific treatment monitoring, as they may provide computational feedback to validate biological hypotheses generated from OoC experiments. In parallel, recent applications of DL to EV-based molecular profiling suggest that EV data could complement chip-derived functional readouts. For example, CNN-based approaches have been used for automated spot detection and co-localization of multi-miRNA signatures in individual EVs, including miR-21, thereby supporting real-time assessment of tumor progression and invasiveness in chip-compatible models [110]. More recently, DL frameworks integrating digital holography and optical tweezers have enabled label-free classification of GBM-derived EVs based on optical features such as refractive index and scattering properties, supporting high-throughput diagnostic and liquid-biopsy applications [111]. Beyond miRNA profiling, circulating GBM EVs contain molecular cargo suitable for computational analysis: serum exosomal miRNA signatures have been used to distinguish GBM patients from controls with machine learning classifiers [112], whereas EV-derived DNA methylation and mutation profiles may reflect tumor subtype and mutational status [113]. Collectively, these findings support the potential integration of EV multi-omic data, OoC-derived functional readouts, and interpretable DL approaches to improve GBM detection, stratification, and longitudinal monitoring [114].

Finally, although reinforcement learning has been explored experimentally in oncology and medical image analysis, its role in treatment optimization remains largely preclinical. Therefore, RL should be presented as a hypothesis-generating approach for simulating adaptive dosing, experimental control, or dynamic treatment-response scenarios within chip systems, rather than as a validated clinical decision-making tool [98]. Emerging technologies such as nanopore sequencing may also become relevant to this framework, as they can support rapid molecular tumor classification and increasingly rely on ML/DL-based signal interpretation. Within an OoC-EV-DL setting, rapid sequencing could provide timely molecular inputs that complement chip-derived functional readouts and EV-cargo profiling, although this remains an area for future validation rather than current clinical implementation.

## 5. Application of Deep Learning in Morphological and Functional Analysis of GBM OOC Models

The literature distinguishes two principal modalities of analysis in the context of OoC models: morphological and functional. However, even with the limited number of studies that focus specifically on OoC systems of GBM, the expanding body of work on DL applications in tumor morphology and function analysis provides a valuable foundation that can be adapted for GBM OoCs. Morphological analysis aims to characterize the structural attributes of tumor cells, including their size, shape, and organization, typically through segmentation and quantification of imaging data. Functional analysis, on the other hand, seeks to capture dynamic behaviors such as proliferation, migration, and response to stimuli, including pharmacological agents. These complementary approaches, when enhanced through DL, offer a powerful means to extract comprehensive insights from complex microphysiological systems [114].

DL techniques, especially CNN architectures like U-Net, have demonstrated significant success in the automated segmentation and morphological characterization of complex 3D cancer models, such as spheroids, which share similarities with tumor structures in OoCs. In practice, DL pipelines have been developed for the precise detection and segmentation of 3D spheroids from microscopy images, achieving high precision (e.g., 95% prediction accuracy reported in a study using U-Net) and allowing the extraction of quantitative morphological features [115]. Variants such as 3D CNNs and fusion networks (combining U-Net and SegNet features) are being explored for detailed volumetric segmentation of GBM in clinical imaging [116], and similar approaches, such as the combination of multiple 2D convolutional networks [62], could be adapted for high-resolution imaging data from GBM-OoCs. These methods allow for objective and high-throughput quantification of morphological parameters (e.g., volume, circularity, texture) that can indicate tumor heterogeneity, growth patterns, or response to microenvironmental cues within the OoC.

Beyond static morphology, DL is increasingly applied to analyze the dynamic functional behavior of cancer cells within OoCs and related models. This includes tracking cell migration, assessing viability, and predicting therapeutic responses. Microfluidic platforms, often used in OoC development, enable live-cell imaging suitable for monitoring processes like GBM cell migration through confined channels, simulating infiltration [117]. DL algorithms can automate the analysis of these time-lapse sequences, quantifying migration speed, directionality, and potentially correlating migratory behavior with cell morphology [118]. Furthermore, DL models, particularly CNNs, are being trained to assess cell viability directly from brightfield or fluorescence images of 3D cultures, offering label-free or minimally invasive methods for monitoring tumor health and response to treatment [119]. Such image-based viability estimations have been used for longitudinal assessment of drug interactions (synergy, antagonism) in GBM spheroid models [120]. Integrating scalable OoC platforms with DL-based image analysis pipelines presents a significant step towards high-throughput functional screening for drug discovery and personalized oncology, enabling the prediction of drug efficacy based on phenotypic changes observed within the chip [121].

In this context, the analysis of EVs through DL can represent an important advancement in completing the functional landscape of the TME, as these particles act as fundamental mediators in intercellular communication and pathological progression. Recently, EV interaction has been studied using segmentation approaches such as Dual-UNet architectures with triple-prediction strategies, enabling objective quantification of internalized EVs, uptake rates, and cellular area coverage through time-lapse sequences [122]. In addition, integrating AI with multi-omics profiling of EVs opens new pathways for personalized medicine and drug discovery. DL models can be trained to decode biogenesis and cargo sorting mechanisms and to predict real-time therapeutic responses through liquid biopsies [123]. In GBM, chip-based analysis has already shown that exosomal mRNA can mediate drug resistance, and recent patient-derived 3D cultures and plasma EV studies illustrate how EV surface markers and multi-omics signatures can support glioma biomarker discovery [64,65,98,124]. These studies represent important precursors to the integrated framework proposed here, even though no end-to-end OoC-EV-DL platform for GBM precision oncology has yet been validated. The integration of scalable OoC platforms with DL-based image analysis pipelines thus constitutes a decisive step toward high-throughput functional screening, enabling the design of engineered EVs for targeted therapies and advanced diagnostics in next-generation precision oncology.

## 6. Predictive Modeling of Drug Response and Personalized Medicine

The integration of OoC technology with DL may help translate descriptive in vitro observations into experimentally testable predictions, although its clinical use remains aspirational. Patient-derived GBM cells cultured in OoC devices can serve as functional testing platforms in which treatment-induced changes in morphology, migration, viability, barrier permeability, immune-cell behavior, imaging features, omics profiles, and EV cargo are measured in a controlled and longitudinal manner. Compared with conventional static cultures, these systems may better preserve relevant aspects of tumor–microenvironment interaction, including vascular-like barriers, soluble-factor exchange, immune modulation, and dynamic EV-mediated communication. When these readouts are integrated into DL pipelines, they may provide a multidimensional representation of tumor behavior before and after therapeutic exposure.

**Figure 1:**
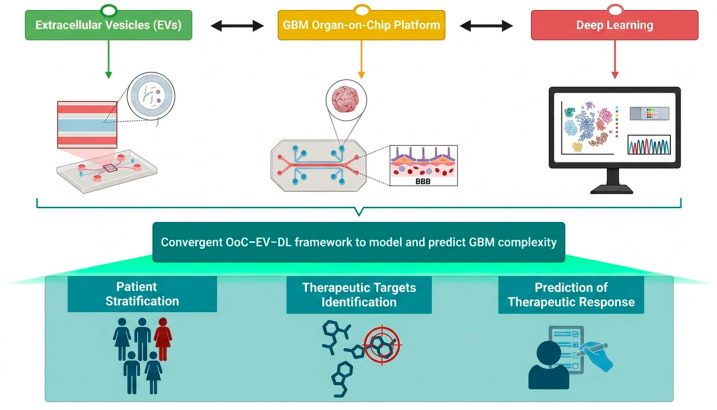
Schematic representation of the integrated OoC–EV–DL framework proposed to decode GBM complexity. The model brings together three tightly interconnected dimensions: (i) investigation of the tumor microenvironment and extracellular vesicles (EVs) as active mediators of intercellular communication; (ii) GBM organ-on-chip platforms capable of dynamically reproducing key features such as the perivascular niche, hypoxic gradients, and blood–brain barrier interactions; and (iii) deep learning approaches for the integrated analysis of morphological, functional, and multi-omics data. The convergence of these elements is proposed as a preclinical experimental framework for modeling tumor heterogeneity, prioritizing candidate biomarkers, and generating hypotheses on treatment response. The image was created using FigureLabs and subsequently edited by the author for clarity and presentation purposes.

Subsequently, DL models can be conditioned to link these extensive biological readouts with an experimental endpoint such as drug sensitivity, resistance, BBB breakdown, invasive potential, or molecular subtype-specific response. This approach could enable scenario testing for individual patients, creating a dynamic interplay between patient information, EV-based molecular data, and functional OoC experimental outcomes, thus constituting a pivotal nexus in experimental neuro-oncology and personalized medicine (Figure 1).

However, these approaches should still be regarded as preclinical decision-support tools until they are prospectively validated, benchmarked against standard assays, and shown to generalize across institutions, experimental platforms, and patient cohorts [97,98,99,100,119,120,121,122,123,124,125].

To the best of our knowledge, existing studies do not yet present a computational framework or DL model that simultaneously integrates OoC and EV data with multi-omics/imaging predictive modeling of therapeutic response in GBM. An emerging direction is therefore to develop multimodal machine learning models for OoC and EV data. These models can incorporate complementary aspects of patient-specific tumor biology by jointly analyzing organoid-derived features (e.g., imaging, metabolic and transcriptomic data) with ligand binding or protein expression profiles of molecularly characterized EVs in clinically relevant conditions. These approaches include multi-branch neural networks, graph-based representations, or feature-level fusion that allow for the integration of various omics data, providing an end-to-end prediction of therapeutic responses to create a potent personalized oncology strategy capitalizing on the strengths of each OoC and EV modality.

## 7. Challenges and Future Directions in Merging Deep Learning with Organ-on-Chip Technology

**Figure 2:**
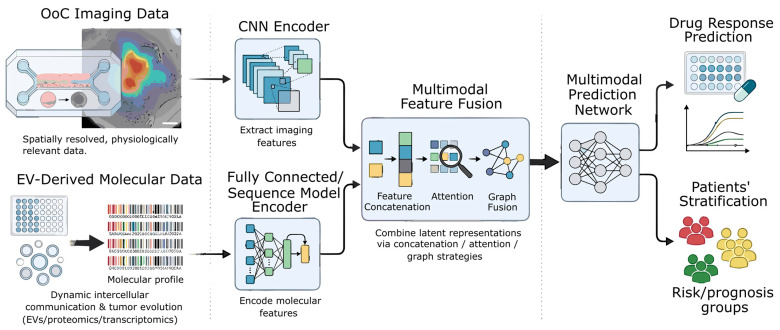
Schematic representation of the proposed multimodal framework integrating organ-on-chip (OoC) systems, extracellular vesicles (EVs), and deep learning (DL) architectures for GBM modeling. OoC-derived imaging, biosensor, barrier-function, and EV-derived molecular data are processed through modality-specific encoders and integrated through multimodal fusion strategies for downstream experimental tasks like drug-response prediction, immune-state classification, and biomarker prioritization. The image was created by the authors using FigureLabs and subsequently edited for clarity and presentation purposes.

The design of OoC platforms and the integration of DL pipelines pose numerous technical and conceptual challenges, such as the lack of standardized, annotated datasets derived from OoC experiments, making it difficult to generate easily reproducible models. Biological complexity represents an additional barrier. The immunosuppressive GBM microenvironment includes GAMs, MDSCs, Tregs, endothelial cells, astrocytes, pericytes, and extracellular matrix components that are difficult to reproduce simultaneously. Future immune-competent GBM-on-chip models should incorporate selected immune components in a modular fashion, allowing investigators to test how GBM-derived EVs reprogram macrophages/microglia, recruit or activate suppressive myeloid populations, impair T-cell function, and interact with BBB elements [6,7,8,9,25,26,27,28,124]. DL models may help distinguish immune-derived from tumor-derived molecular signals by integrating lineage-aware imaging, surface-marker profiles, single-cell or spatial omics, and EV-cargo data. More broadly, a key future direction is the development of multimodal architectures capable of integrating OoC-derived imaging, biosensor and barrier-function readouts with EV-derived molecular profiles. In such a framework, modality-specific encoders could process imaging, functional, and molecular inputs separately, while multimodal fusion strategies, including feature concatenation, attention-based fusion, or graph-based integration, could generate shared latent representations for downstream tasks such as drug-response prediction, immune-state classification, and biomarker prioritization (Figure 2). However, these computational distinctions must be validated experimentally rather than inferred from model outputs alone.

A realistic translational roadmap should also take into account other considerations such as cost, throughput, regulatory barriers and even computational bias. The demand for OoC platforms can incur high costs and technical complexities and also be challenging to scale at the level of thousands of patients. EV isolation and characterization are still affected by pre-analytical variables, and to avoid all bias with respect to institution, acquisition protocol, patient selection or molecular subtype, DL models require representative datasets. Consequently, regulatory translation would have to involve reproducible chip manufacturing, standardized analytical pipelines, quality-control thresholds, locked computational models, auditable decision rules and prospective evidence that the framework improves clinically meaningful outcomes. We expect that mechanistic studies, biomarker prioritization and preclinical drug-screening workflows will be near-term applications, while the use of this knowledge for routine clinical treatment selection should be considered a longer-term goal.

In conclusion, the convergence of OoC systems, the biology of EVs, and DL offers a strategy that is highly preliminary but nevertheless promising for investigating GBM complexity. The greatest strength of this analytical framework is that controlled experimental perturbations can be seamlessly associated with spatial, functional and molecular readouts. Its major limitations include a lack of large datasets, uncertainty in EV source attribution, incomplete immune and BBB modeling, risks of overfitting and domain shift, and many unresolved regulatory- and scalability-related issues. Overcoming these hurdles will be critical to advancing OoC-EV-DL platforms from conceptual frameworks to validated modalities for precision-oriented GBM research.

## Data Availability

Not applicable.
